# Growth of Very Preterm Infants in a Low-Resourced Rural Setting after Affiliation with a Human Milk Bank

**DOI:** 10.3390/children9010080

**Published:** 2022-01-05

**Authors:** Chia-Huei Chen, Hui-Ya Chiu, Szu-Chia Lee, Hung-Yang Chang, Jui-Hsing Chang, Yen-Ju Chen, Lin Kang, Shang-Po Shen, Yung-Chieh Lin

**Affiliations:** 1 Department of Pediatrics, Taitung MacKay Memorial Hospital, Taitung 950408, Taiwan; ayacm@hotmail.com (C.-H.C.); immilu@hotmail.com (H.-Y.C.); latte_azure@hotmail.com (S.-C.L.); 2 Division of Neonatology, Department of Pediatrics, MacKay Children’s Hospital, Taipei 104217, Taiwan; 4583@mmh.org.tw (H.-Y.C.); jhchang90@yahoo.com.tw (J.-H.C.); 3 Department of Pediatrics, National Cheng Kung University Hospital, College of Medicine, National Cheng-Kung University, Tainan 704302, Taiwan; yensweet@gmail.com; 4 Taiwan Southern Human Milk Bank, National Cheng Kung University Hospital, College of Medicine, National Cheng-Kung University, Tainan 704302, Taiwan; kanglin@mail.ncku.edu.tw; 5 Department of Obstetrics and Gynecology, National Cheng Kung University Hospital, College of Medicine, National Cheng-Kung University, Tainan 704302, Taiwan

**Keywords:** preterm infants, extrauterine growth restriction, human milk bank, nutrition protocol, quality improvement

## Abstract

The extrauterine growth restriction (EUGR) of very preterm infants has been associated with long-term complications and neurodevelopmental problems. EUGR has been reported at higher rates in low resource settings. There is limited research investigating how metropolitan human milk banks contribute to the growth outcomes of very preterm infants cared in rural areas. The setting of this study is located at a rural county in Taiwan and affiliated with the Taiwan Southern Human Milk Bank. Donor human milk was provided through a novel supplemental system. A renewal nutritional protocol was initiated as a quality improvement project after the affiliated program. This study aimed to compare the clinical morbidities and growth outcome at term equivalent age (TEA) of preterm infants less than 33 weeks of gestational age before (Epoch-I, July 2015–June 2018, *n* = 40) and after the new implementation (Epoch-II, July 2018–December 2020, *n* = 42). The Epoch-II group significantly increased in bodyweight z-score at TEA ((−0.02 ± 1.00) versus Epoch-I group (−0.84 ± 1.08), *p* = 0.002). In multivariate regression models, the statistical difference between two epochs in bodyweight z-score changes from birth to TEA was still noted. Modern human milk banks may facilitate the nutritional protocol renewal in rural areas and improve the growth outcomes of very preterm infants cared for. Establishing more distribution sites of milk banks should be encouraged.

## 1. Introduction

Extrauterine growth restriction (EUGR), defined as an anthropometric measure that is lower than the 10th percentile, is prevalent and occurs in the majority of very preterm infants [1]. Preterm infants with EUGR at discharge have been shown to develop short stature, poor neurodevelopmental outcomes, and cardiovascular and metabolic problems after they grow up [2,3,4,5,6]. EUGR has been linked to intrauterine growth restriction, maternal preeclampsia, low gestational age (GA), feeding intolerance, delay in first enteral feeding, and inadequate nutritional supplement [1,7,8,9,10,11].

To minimize the prevalence of EUGR, standardized nutritional protocols have been proposed in recent years. Early trophic feeding (ETF) and optimal parenteral nutrition (PN) supplements have been recommended to be implemented in every neonatal intensive care unit (NICU) by several studies [12,13,14,15,16,17]. However, in practice, discrepancies between city and rural institutions have been largely overlooked despite the well-known benefits of ETF reported [18,19,20]. In rural hospitals, enteral feeding could be delayed due to the fear of necrotizing enterocolitis (NEC) and the shortage of pediatric surgeons [13,15,21]. The accessibility of a human milk bank (HMB) could be an important factor associated with initiating ETF. The benefits of implementation of an HMB in NICUs have been reported by several studies (Table S1) [22,23,24,25,26]. To date, seldom studies report the influence on growth outcome of these very preterm infants after the implementation of an HMB in NICUs.

Our institution is located at a low-resource area in Taiwan, and the accessibility of donor human milk (DHM) had been difficult for years. However, since July 2018, we have been the first distribution site of the Taiwan Southern Human Milk Bank (TSHMB) (Table S2). Under a novel cooperation model (see methodology Section 2.3), DHM was readily provided without any cost. This cooperation also provides an opportunity to implement a renewal nutrition protocol to fit the recommendations of the ETF guideline of the Taiwan Society of Neonatology [27].

This study primarily aimed to review the growth outcome of very preterm infants at term equivalent age (TEA, post-menstrual age 37 to 42 weeks) before and after the quality improvement (QI) of the renewal nutritional protocol related to the affiliation with an HMB [28]. This study also investigated the effectiveness and safety of the implementation as the secondary outcome.

## 2. Materials and Methods

### 2.1. Study Design

We conducted this retrospective study and included all preterm infants less than 33 weeks of GA born between July 2015 and December 2020. Infants with congenital disease or major congenital anomalies involving the cardiovascular or gastrointestinal system were excluded.

Considering the basis of the nutritional protocol before and after the QI project implementation, enrolled infants were separated into two groups for historical comparison: Epoch-I group (July 2015–June 2018) and Epoch-II group (July 2018–December 2020). The study was approved by the Institutional Review Board of MacKay Memorial Hospital (IRB approval number: 21MMHIS079e).

### 2.2. Study Setting and Affiliation with Human Milk Bank

This study was conducted at a seven-bed NICU at Taitung MacKay Memorial Hospital in Taitung County, Taiwan. Taitung is a rural county located in the east-south of Taiwan Island.

Approximately 70 neonates were treated at the unit yearly, including approximately 15 infants with a very low birth weight. One neonatologist, one resident, and one nurse practitioner were regularly in charge of the patients. The unit was equipped with a milk preparation room.

Since July 2018, this unit began affiliating with TSHMB, which is 200 km from the unit. The first small distribution site of pasteurized DHM was set up. DHM is always ready to be used for newborns whose mothers’ own milk (MOM) is insufficient or unavailable. The families are requested to sign consent for using DHM and the cost is supported by a government project of the Ministry of Health and Welfare, Taiwan. When there is a shortage of DHM in the unit, a new supply will arrive within 24 h after placing an order—orders can be conveniently made through Line^®^, a social network service (Table S2). The DHM delivery are through a convenient cold chain network operated by a Taiwan private company (SEVEN-ELEVEN ^®^, Taiwan), which also owned more than 6000 stores by 2021. A shipment usually includes an ice bucket (Dometic^®^ WCI-33, Sweden) with around 50 bottles (100 cc DHM/bottle).

### 2.3. Nutrition Protocol

The brief comparisons between the two epochs are listed in Appendix A Table A1. The details of the implemented protocol are explained in the following subsection.

#### 2.3.1. Parental Nutrition

The standardized nutritional protocol, modified based on recommendations from the Taiwan Society of Neonatology, started the PN soon after the patient was born, targeting 3–3.5 g per kilogram per day (g/kg/d) of protein by the first day of life (DOL) and increasing to 4 g/kg/d. Lipids were administrated at 1 g/kg/d by first or second DOL and increased to 3 g/kg/d with an increment rate of 0.5–1 g/kg/d. The dextrose concentrations were started at a glucose infusion rate of 4.8 milligrams per kilogram per minute (mg/kg/min) and then increased by daily fluid requirement.

The calcium and phosphorus content in PN was adjusted to the proper 1.3:1 molar ratio to reach the recommendation of the American Academy of Pediatrics.

PN was ceased once the feeding amount reached 120 milliliters per kilogram per day (mL/kg/d) if there was no further indication for intravenous fluid administration.

#### 2.3.2. Enteral Nutrition

The protocol emphasized the use of human milk, including MOM and DHM, and the practice of ETF. Owing to the limited supplement of DHM, patients who were born at less than 30 weeks of gestational age had the top priority to access DHM if MOM was unavailable. Those that were born between 31 to 33 weeks of gestational age whose MOM was unavailable had the next priority. Only those who had unstable clinical condition delayed the initiation of enteral feeding. The volume of ETF was set at 15 mL/kg/d. The length of ETF was set according to the birth weight of the patient. Five-day ETF was set for those with birth weights less than 700 gm, 3-day for those with birth weight between 701 gm and 1000 gm, and 2-day for those with birth weights between 1001 gm and 1250 gm. After ETF was accomplished, the rate of incensement was set at 20 mL/kg/d per day. The feedings were interrupted, or workups were implemented if one of the following was present: (1) bilious or bloody residuals, (2) existing peritoneal signs on physical examination, (3) respiratory problems probably related to feeding. Two-staged fortification was applied, and we fortified human milk with human milk fortifier or concentrated preterm formula [29]. First (22 Kcal/oz) and second (24 Kacl/oz) fortifications were performed after the feeding amount reached 100–120 and 150 mL/kg/d. The increment of feeding amount would be held for 3 days after the first fortification.

### 2.4. Outcomes and Data Collection

The variables collected from maternal and neonatal electronic chart included perinatal history, the day of nutrition intervention after admission, length of hospital stay, anthropometrical measurement at TEA, medication history, and neonatal morbidities during hospitalization. The GA of a fetus was calculated from the established date of delivery according to the last menstrual period.

The primary outcome data were the Δz-score of body weight (BW) at TEA, the difference of bodyweight z-score between birth and TEA. We calculated the z-scores and percentiles on the basis of reference data on the website [30]. The secondary outcomes were the nutritional practice and the major neonatal morbidity during hospitalization including bronchopulmonary dysplasia (BPD), treated retinopathy of prematurity (ROP), NEC ≥ stage IIA, and late-onset sepsis (LOS) [31,32,33].

### 2.5. Statistics and Analysis

All analyses were performed using SPSS (Version 26, IBM, Armonk, NY, USA). A *p*-value of 0.05 was considered significant. We compared continuous variables with Student’s *t*-test or Mann–Whitney’s U test, whereas we compared categorical data with the chi-square test or Fisher’s exact test, where applicable. The outcome of infants was compared between the study periods utilizing the analysis of covariance. In analysis models, covariates were selected with *a priori* for clinical consideration.

## 3. Results

**Figure 1:**
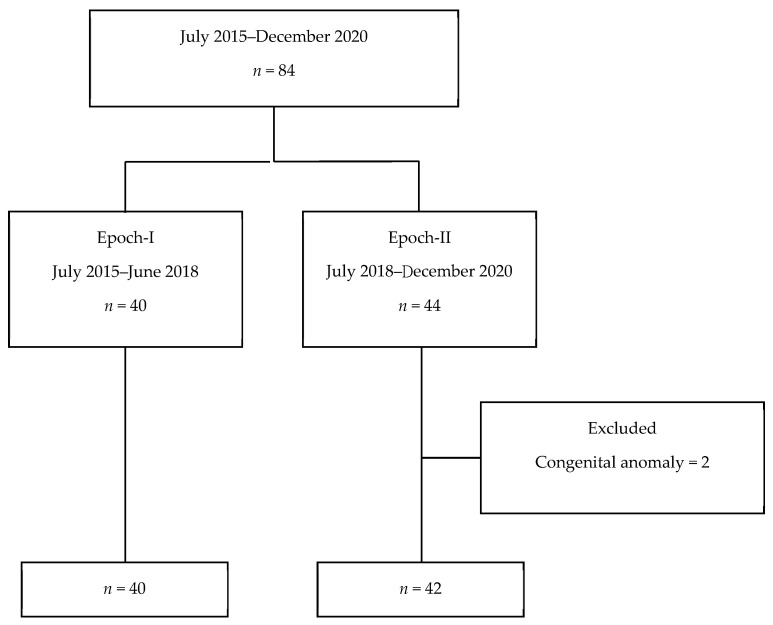
Flow chart of patient enrollment. *n*: case number.

Eighty-four preterm infants were included during the study period, and excluded infants included one extensive Klippel–Trenaunay syndrome and one congenital complex heart disease. (Figure 1). Among these 82 neonates enrolled in the analysis, 40 were in the Epoch-I group and 42 were in the Epoch-II group.

The Epoch-II group had earlier administration of PN, first enteral feeding, milk fortification, and higher exclusive MOM or DHM feeding before fortification than the Epoch-I group. The length of PN administration was shorter in the Epoch-II group (Appendix A Table A1).

The GA was 28.7 ± 2.4 and 28.1 ± 2.6 (weeks, *p* = 0.229) and birth weight was 1120 ± 363 and 1132 ± 360 (grams, *p* = 0.912) in Epoch-I and Epoch-II groups, respectively. The Epoch-I group had lower z-scores of birth weight (−0.32 ± 0.93 vs. 0.07 ± 0.71, *p* = 0.033), higher pH of 1st blood gas (7.29 ± 0.96 vs. 7.24 ± 0.91, *p* = 0.008), and higher rate of small for gestational age (20.0% vs. 2.4%, *p* = 0.007), maternal absent or reverse-end diastolic velocity (20.0% vs. 2.4%, *p* = 0.007), and usage of umbilical line (20.0% vs. 4.8%, *p* = 0.035). Otherwise, there was no significant difference between the two groups in baseline demographic characteristics (Table 1).

Seven patients in each group were excluded from the growth outcome analysis because of inaccessibility of BW at TEA (Epoch-I: four died, two were transferred to other institutions, one was lost to follow up; Epoch-II: five died, two were lost to follow up). The mean follow-up rate was 82.9%.

Although both groups shared similar post-menstrual ages at discharge and lengths of hospital stay, the Epoch-I group had lower BWs (3217 ± 552 vs. 3572 ± 448, grams, *p* = 0.006) and z-scores for BW (−0.84 ± 1.08 vs. −0.02 ± 1.00, *p* = 0.002) at TEA.

Factors which may be associated with the growth outcomes were listed and analyzed (Table 2). Infants with higher gestational age were related to better Δz-score of body weight (mean coefficient = 0.198; 95% confidence interval [CI]: 0.098–0.298, *p* < 0.001). Neonatal morbidities had significantly negative association with Δz-score of body weight at TEA, including PDA ligation (mean coefficient = −0.957, 95% CI: −1.705–−0.209, *p* = 0.013) and bronchopulmonary dysplasia (mean coefficient = −0.659, 95% CI: −1.156–−0.162, *p* = 0.010).

Although there was no statistical significance between both epochs in the univariate model in regards to the Δz-score of BW (mean coefficient 0.347, 95% CI: −0.122–0.816, *p* = 0.145) (Table 2), the differences were noted significantly after multivariate linear regression (Table 3, models 1 and 2). In the model 3, epoch had borderline significance in a positive association with Δz-score of BW, which was adjusted with one of neonatal morbidities, PDA ligation (mean coefficient = 0.429, 95% CI: −0.016–0.874, *p* = 0.058) (Table 3). In the model 3, the *p* value of epoch was more significant than the *p* values of PDA ligation, which may hint the effect of epoch may be a more important factor than neonatal morbidities related to Δz-score of BW. Similar results were observed when selecting late onset sepsis or bronchopulmonary dysplasia into multivariate models (Table S5).

We further tested using absolute BW and z-score of BW at TEA separately as primary growth outcomes. Epoch played significantly important roles in both dependent variables after multivariate linear regression (Tables S3 and S4).

For secondary outcomes, no statistical difference was noted between the two epochs on the aspect of death, NEC ≥ IIA, treated ROP, and BPD. The prevalence of LOS was higher in the Epoch-I group (31% vs. 2.4%, *p* < 0.001) (Table 1).

## 4. Discussion

In this QI project coordinated with an HMB, we found a significant decreasing prevalence of EUGR and Δz-score of BW of very preterm infants at TEA after implementing a standardized nutritional protocol with intensive nutritional supplementation and ETF. We also found a markedly decreased prevalence of LOS without an increase in the death rate or NEC for those receiving the new nutritional protocol. To our best knowledge, the present study is the first that discusses the effectiveness and safety of the standardized nutritional protocol that is held in a rural neonatal intensive care unit with a novel utilization of HMB.

### 4.1. The Influence of DHM Accessibility on Feeding Policy

The increased accessibility of DHM after affiliation with HMB may impact the feeding policy [34]. The first impact may be related to age of initiation of feeding (Appendix A Table A1). During Epoch-II, most of the preterm babies were routinely fed on the day of their birth unless the clinical condition was unstable based on the new nutritional protocol. Only one patient who received postnatal resuscitation due to perinatal asphyxia did not meet the criteria for early enteral feeding. Physicians fed the preterm infants earlier in Epoch-II —3.3 days earlier than Epoch-I. The very preterm infants of Epoch-II reached full enteral feeding earlier—19.5 days earlier than those in Epoch-I. Our findings were consistent with previous studies reporting that the nutritional practices directly related to EUGR included age at first enteral feeding and full enteral feeding, and early feeding is related to early achievement of full enteral feeding [35].

Higher rates of exclusive MOM or DHM feeding before fortification were noted in the Epoch-II group. This resulted from the cooperation between our institute and TSHMB since July 2018. Since then, almost all very low birth weight infants have been fed with their MOM or DHM in our unit. Previous studies reported that using MOM and DHM decreased the incidence of feeding intolerance and the risk of NEC [36]. Although we did not observe a decrease in the rate of NEC between two epochs, the use of MOM and DHM did facilitate our medical staff, including attending physicians, pediatric residents, and nurses, to enforce the new feeding protocol.

### 4.2. Renewal of Nutritional Protocol, Growth Outcomes, and Neonatal Morbidities

In this study, z-score of BW, Δz-score of BW, and presence of EUGR at TEA reached statistical difference between the two groups. Although the significance of the presence of EUGR disappeared after logistic regression analysis, the statistical differences of z-score of BW, Δz-score of BW at TEA persisted after multivariate linear regression analysis. The results were compatible with previous studies that showed a standardized nutritional protocol could reduce the incidence of EUGR in preterm infants [12,15]. The lower Δz-score of BW at TEA could explain why the patients in Epoch-II had a higher z-score of BW at TEA while comparing with those in Epoch-I.

In addition, we did not interrupt the feeding based on the gastric residual volume, since current evidence showed that routine checking of the gastric residual volume would delay the time of reaching full enteral feeding without benefits for preventing NEC [37]. This may also be a reason why the time to reach full enteral feeding decreased so dramatically after implementing the standardized nutritional protocol. Along with a shorter time to reach full enteral feeding, the length of PN administration became shorter, and the rate of central line usage and LOS became lower.

### 4.3. The Benefits, Cost, and Challenge of HMB during COVID-19 Pandemic

NEC has been related to high medical cost [38]. Fortunately, HMB has been reported to increase human milk availability in reginal or community NICUs and has been associated with a decrease in the NEC rate [34]. However, those studies seldom reported the growth outcome after the increased availability of human milk from milk banks. Our study has linked the HMB affiliation and a renewal of nutrition protocol. In addition, our study has shown the improvement of growth outcome in very preterm infants without increasing NEC rate. Our beneficial findings may help to promote the establishment of affiliation sites of HMB for the debate of the high cost of DHM [39]. After further analysis (not shown in tables), the cost of DHM in this study was 2–3 US$ per ounce, which was much lower than 3–5 US$ per ounce reported in other studies [39,40]. Importantly, the rate of persistent MOM feeding after discharge non-significant increased from 36.4% to 45.7% after affiliation with the HMB (Table 1), which has been reported in the work of Delfosse [41].

Moreover, it is worth noting that the study period of Epoch-II was during the COVID-19 pandemic. Despite the persistent use of MOM after discharge, which did yield statistical significance between the two groups, we did observe a higher portion of MOM use in Epoch-II after the patient was discharged. It has been reported that DHM supplementation could increase the rate of exclusive breastfeeding [22,42]. Considering the benefits of MOM to long-term neurodevelopmental outcomes, maintaining an adequate supplement of safe DHM during the pandemic became a major issue [43].

### 4.4. Limitations

This study had several limitations. First, as with many comparisons with historical cohorts, the main limitation of the study is the possibility that changes in management of infants other than HDM may influence the outcomes. For example, the initial PN was started earlier in Epoch-II than in Epoch-I. Under limited case number in this rural hospital, we only could investigate with historical cohorts to explain the effect of implanting “a bundle” of changes after affiliation of a human milk bank. Changes compatible with the implantation were also observed, including shorter duration of total parenteral nutrition, earlier age of initial feeding and full feeding, more infants received fortified human milk not fortified formula, less umbilical lines, less peripheral inserted lines, and less catheter related late onset sepsis.

Second, the small sample size of preterm infants enrolled in the trial was relatively small; we could not control more potential confounders in the multivariate analysis to see if the Δz-score of body weight TEA is associated with the epoch. However, the epoch could explain significant associations with the growth in some results of the many multivariate models we tested. To overcome these disadvantages, a large population-based, multi-institution prospective study should be conducted in the future.

Third, there was a lack of data regarding the exact daily caloric intake and other anthropometrical outcomes, such as head circumference and body length at TEA.

## 5. Conclusions

A renewed nutrition protocol coordinated with an HMB may improve the growth outcomes of very preterm infants born in rural areas. Future research should continue to explore the effects of distribution sites of the modern HMB in multi-hospitals.

## Figures and Tables

**Table 1:** Baseline demographic characteristics.

	*n* for Analysis	Epoch-I (*n* = 40)	Epoch-II (*n* = 42)	*p*-Value
***Perinatal variables***				
Gestational age, weeks	82	28.7 ± 2.4	28.1 ± 2.6	0.229
Birth weight, grams	82	1120 ± 363	1132 ± 360	0.912
Z-score of birth weight	82	−0.32 ± 0.93	0.07 ± 0.71	**0.033**
Small for gestational age	82	8 (20.0%)	1 (2.4%)	**0.007**
Gender: male	82	26 (65.0%)	26 (61.9%)	0.771
Cesarean delivery	82	21 (52.5%)	20 (47.6%)	0.659
AEDV/REDV	82	8 (20.0%)	1 (2.4%)	**0.007**
Multi-gestational pregnancy	82	4 (10.0%)	10 (23.8%)	0.097
Antenatal steroid	82	34 (85.0%)	36 (85.7%)	0.927
***Neonatal variables*** APGAR score				
1st minute, median (range)	82	7 (2–9)	7 (1–10)	0.167
5th minute, median (range)	82	9 (5–10)	9 (2–10)	0.111
pH of 1st blood gas analysis	82	7.29 ± 0.96	7.24 ± 0.91	**0.008**
Surfactant administration	82	16 (40%)	11 (26.2%)	0.183
Umbilical line	82	8 (20%)	2 (4.8%)	**0.035**
hsPDA requiring surgery	82	7 (17.5%)	3 (7.1%)	0.185
Late onset sepsis	80 ^1^	12 (30.8%)	1 (2.4%)	**<0.001**
Necrotizing enterocolitis ≥ IIA	80 ^1^	3 (7.7%)	3 (7.3%)	1.000
Treated retinopathy of prematurity	73 ^2^	2 (5.3%)	6 (16.2%)	0.279
Bronchopulmonary dysplasia	73 ^2^	15 (41.6%)	9 (24.3%)	0.115
Death	82	4 (10%)	5 (11.9%)	0.938
Discharge PMA, weeks	70 ^3^	38.5 ± 3.0	38.5 ± 5.1	0.987
Length of hospital stay, days	70 ^3^	67.1 ± 30.0	72.1 ± 47.1	0.600
Z-score of body weight at TEA	68 ^4^	−0.84 ± 1.08	−0.02 ± 1.00	**0.002**
EUGR	68 ^4^	11 (33%)	4 (11.4%)	**0.029**
Persistent MOM feeding after discharge (with or without mixing with PDF)	68 ^4^	12 (36.4%)	16 (45.7%)	0.434

Data are presented as the mean ± standard deviation or number (percentage). *n*: case number; AEDV: absence of end diastolic velocity; REDV: reverse of end diastolic velocity; hsPDA: hemodynamically significant patent ductus arteriosus; PMA: post-menstrual age; TEA: term equivalent age; EUGR: extrauterine growth restriction; MOM: mother’s own milk; PDF: post-discharge formula. ^1^ One patient died at the same day admitted to NICU in each group was excluded from analysis. ^2^ Deaths were excluded from analysis. ^3^ Seven patients in Epoch-I and 5 patients in Epoch-II died or transferred to other hospital were excluded from analysis. ^4^ Seven patients in Epoch-I and 7 patients in Epoch-II died, transferred to other hospital, or lost to follow up were excluded from analysis. Statistical significance was assumed for *p* < 0.05 (indicated in bold).

**Table 2:** Dependence of Δz-score of body weight at term equivalent age on clinical variables: univariate analysis.

	Univariate		
	B	95%CI (LB, UB)	*p*-Value
Gestational age, week	0.198	(0.098, 0.298)	**<0.001**
Small for gestational age	0.292	(−0.544, 1.129)	0.488
Z-score of birth weight	−0.234	(−0.527, 0.060)	0.117
Sex	0.331	(−0.146, 0.808)	0.171
Cesarean section	0.024	(−0.453, 0.510)	0.920
Antenatal steroid	−0.106	(−0.778, 0.567)	0.755
Multi-gestational pregnancy	0.587	(−0.079, 1.244)	0.079
PDA ligation	−0.957	(−1.705, −0.209)	**0.013**
Bronchopulmonary dysplasia	−0.659	(−1.156, −0.162)	**0.010**
Late onset sepsis	−0.580	(−1.217, 0.056)	0.073
Epoch (ref. Epoch I)	0.347	(−0.122, 0.816)	0.145

Linear regression was performed for each independent variable. Statistical significance was assumed for *p* < 0.05 (indicated in bold). PDA: patent ductus arteriosus; B: mean of coefficients; CI: confidence interval; LB: lower border; UB: upper border.

**Table 3:** Dependence of Δz-score of body weight at term equivalent age on clinical variables: multivariate analyses.

	Multivariate Model 1			Multivariate Model 2			Multivariate Model 3		
	B	95%CI (LB, UB)	*p*	B	95%CI (LB, UB)	*p*	B	95%CI (LB, UB)	*p*
Gestational age	0.204	(0.102, 0.306)	**<0.001**	0.200	(0.099, 0.301)	**<0.001**	0.189	(0.084, 0.295)	**0.001**
Z-score of birth weight	−0.123	(−0.406, 0.161)	0.392	−0.130	(−0.411, 0.150)	0.356	−0.122	(−0.404, 0.160)	0.389
Sex	0.373	(−0.047, 0.794)	0.081	0.339	(−0.078, 0.756)	0.109	0.320	(−0.101, 0.742)	0.133
Multi-gestational Pregnancy				0.481	(−0.095, 1.057)	0.100	0.456	(−0.126, 1.037)	0.122
PDA ligation							−0.276	(−1.014, 0.461)	0.457
Epoch (ref. Epoch I)	0.501	(0.073, 0.929)	**0.022**	0.479	(0.056, 0.902)	**0.027**	0.429	(−0.016, 0.874)	0.058

Multivariate linear regression was performed for each dependent variable. Statistical significance was assumed for *p* < 0.05 (indicated in bold). PDA: patent ductus arteriosus; B: mean of coefficients; CI: confidence interval; LB: lower border; UB: upper border.

## Data Availability

The data presented in this study are available on request from the corresponding author after submitting application to Institutional Review Board of MacKay Memorial Hospital. The data are not publicly available due to the restriction of our Institutional Review Board.

## Supplementary Materials

The following are available online at https://www.mdpi.com/article/10.3390/children9010080/s1, Table S1. Mini reviews: Summarized benefits of the implementation of human milk bank in neonatal intensive care units. Table S2. Characteristics and novelties of Taiwan Southern Human Milk Bank. Table S3. Dependence of body weight at term equivalent age on clinical variables (univariate analysis and multivariate analyses). Table S4. Dependence of Z-score of body weight at term equivalent age on clinical variables (univariate analysis and multivariate analyses). Table S5. Dependence of ΔZ-score of body weight at term equivalent age on clinical variables (multivariate analyses).

## Appendix A

**Table A1:** Comparison of nutritional practices in two epochs.

	Epoch-I (*n* = 39) ^1^	Epoch-II (*n* = 41) ^1^	*p*-Value
*Age of initial nutrition management, days*			
PN	1.13 ± 0.74	0.22 ± 0.42	**<0.001**
First enteral feeding	3.27 ± 3.75	0.02 ± 0.16	**<0.001**
*Milk type at first feeding* ^2^			**<0.001**
MOM	5 (13.5%)	1 (2.5%)	
DHM	0 (0%)	32 (80%)	
Preterm formula	32 (86.5%)	7 (17.5%)	
*Fortification initiating* ^3^			
Fortification with exclusive MOM or DHM	5 (13.5%) ^2^	31 (77.5%) ^2^	**<0.001**
Post-menstrual age, weeks	31.8 ± 2.4	30.0 ± 2.0	**0.008**
Postnatal age, days	19.1 ± 16.1	10.9 ± 4.0	**0.003**
*Length of PN use, days*	28.4 ± 14.6	8.0 ± 4.9	**<0.001**
*Feeding amount (with or without fortification)* *reached 120* *mL/kg/day, days*	30.1 ± 14.0	10.6 ± 3.7	**<0.001**
*PICC insertion*	29 (74.4%)	10 (25.6%)	**<0.001**

Data are presented as the mean ± standard deviation or number (percentage). PN: parental nutrition; MOM: mother’s own milk; DHM: donor human milk; PICC: peripherally inserted central catheter. ^1^ One patient died on the same day admitted to NICU in each group was excluded from analysis. ^2^ Two patients in Epoch-I and one patient in Epoch-II died before starting enteral feeding were ^3^ Fortifications mean shifting 20 kcal/cc to higher caloric density. Statistical significance was assumed for *p* < 0.05 (indicated in bold).

## References

[1] Radmacher P.G., Looney S.W., Rafail S.T., Adamkin D.H. (2003). Prediction of extrauterine growth retardation (EUGR) in VVLBW infants. J. Perinatol..

[2] Chien H.C., Chen C.H., Wang T.M., Hsu Y.C., Lin M.C. (2018). Neurodevelopmental outcomes of infants with very low birth weights are associated with the severity of their extra-uterine growth retardation. Pediatr. Neonatol..

[3] Martinez-Jimenez M.D., Gomez-Garcia F.J., Gil-Campos M., Perez-Navero J.L. (2020). Comorbidities in childhood associated with extrauterine growth restriction in preterm infants: A scoping review. Eur. J. Pediatr..

[4] Ordonez-Diaz M.D., Perez-Navero J.L., Flores-Rojas K., Olza-Meneses J., Munoz-Villanueva M.C., Aguilera-Garcia C.M., Gil-Campos M. (2020). Prematurity with Extrauterine Growth Restriction Increases the Risk of Higher Levels of Glucose, Low-Grade of Inflammation and Hypertension in Prepubertal Children. Front. Pediatr..

[5] Pampanini V., Boiani A., De Marchis C., Giacomozzi C., Navas R., Agostino R., Dini F., Ghirri P., Cianfarani S. (2015). Preterm infants with severe extrauterine growth retardation (EUGR) are at high risk of growth impairment during childhood. Eur. J. Pediatr..

[6] Takayanagi T., Shichijo A., Egashira M., Egashira T., Mizukami T. (2019). Extrauterine growth restriction was associated with short stature and thinness in very low birthweight infants at around six years of age. Acta Paediatr..

[7] Zhao X., Ding L., Chen X., Zhu X., Wang J. (2020). Characteristics and risk factors for extrauterine growth retardation in very-low-birth-weight infants. Medicine.

[8] Liao W.L., Lin M.C., Wang T.M., Chen C.H., Taiwan Premature Infant Follow-up N. (2019). Risk factors for postdischarge growth retardation among very-low-birth-weight infants: A nationwide registry study in Taiwan. Pediatr. Neonatol..

[9] Hu F., Tang Q., Wang Y., Wu J., Ruan H., Lu L., Tao Y., Cai W. (2019). Analysis of Nutrition Support in Very Low-Birth-Weight Infants With Extrauterine Growth Restriction. Nutr. Clin. Pract..

[10] Gidi N.W., Goldenberg R.L., Nigussie A.K., McClure E., Mekasha A., Worku B., Siebeck M., Genzel-Boroviczeny O., Muhe L.M. (2020). Incidence and associated factors of extrauterine growth restriction (EUGR) in preterm infants, a cross-sectional study in selected NICUs in Ethiopia. BMJ Paediatr. Open.

[11] Lin Y.C., Lin Y.J., Lin C.H. (2011). Growth and neurodevelopmental outcomes of extremely low birth weight infants: A single center’s experience. Pediatr. Neonatol..

[12] Stevens T.P., Shields E., Campbell D., Combs A., Horgan M., La Gamma E.F., Xiong K., Kacica M. (2018). Statewide Initiative to Reduce Postnatal Growth Restriction among Infants <31 Weeks of Gestation. J. Pediatr..

[13] McKenzie B.L., Edmonds L., Thomson R., Haszard J.J., Houghton L.A. (2018). Nutrition Practices and Predictors of Postnatal Growth in Preterm Infants During Hospitalization: A Longitudinal Study. J. Pediatr. Gastroenterol. Nutr..

[14] Lin Y.H., Hsu Y.C., Lin M.C., Chen C.H., Wang T.M. (2020). The association of macronutrients in human milk with the growth of preterm infants. PLoS ONE.

[15] Darrow M.C.J., Li H., Prince A., McClary J., Walsh M.C. (2019). Improving extrauterine growth: Evaluation of an optimized, standardized neonatal parenteral nutrition protocol. J. Perinatol..

[16] Baillat M., Pauly V., Dagau G., Berbis J., Boubred F., Fayol L. (2021). Association of First-Week Nutrient Intake and Extrauterine Growth Restriction in Moderately Preterm Infants: A Regional Population-Based Study. Nutrients.

[17] Alburaki W., Yusuf K., Dobry J., Sheinfeld R., Alshaikh B. (2021). High Early Parenteral Lipid in Very Preterm Infants: A Randomized-Controlled Trial. J. Pediatr..

[18] Klingenberg C., Embleton N.D., Jacobs S.E., O’Connell L.A., Kuschel C.A. (2012). Enteral feeding practices in very preterm infants: An international survey. Arch. Dis. Child. Fetal Neonatal Ed..

[19] Stevens T.P., Shields E., Campbell D., Combs A., Horgan M., La Gamma E.F., Xiong K., Kacica M. (2016). Variation in Enteral Feeding Practices and Growth Outcomes among Very Premature Infants: A Report from the New York State Perinatal Quality Collaborative. Am. J. Perinatol..

[20] Nakubulwa C., Musiime V., Namiiro F.B., Tumwine J.K., Hongella C., Nyonyintono J., Hedstrom A.B., Opoka R. (2020). Delayed initiation of enteral feeds is associated with postnatal growth failure among preterm infants managed at a rural hospital in Uganda. BMC Pediatr..

[21] Jadcherla S.R., Dail J., Malkar M.B., McClead R., Kelleher K., Nelin L. (2016). Impact of Process Optimization and Quality Improvement Measures on Neonatal Feeding Outcomes at an All-Referral Neonatal Intensive Care Unit. JPEN J. Parenter. Enteral Nutr..

[22] Arslanoglu S., Moro G.E., Bellu R., Turoli D., De Nisi G., Tonetto P., Bertino E. (2013). Presence of human milk bank is associated with elevated rate of exclusive breastfeeding in VLBW infants. J. Perinat. Med..

[23] Sparks H., Linley L., Beaumont J.L., Robinson D.T. (2018). Donor milk intake and infant growth in a South African neonatal unit: A cohort study. Int. Breastfeed. J..

[24] Alyahya W., Barnett D., Cooper A., Garcia A.L., Edwards C.A., Young D., Simpson J.H. (2019). Donated human milk use and subsequent feeding pattern in neonatal units. Int. Breastfeed. J..

[25] Hosseini M., Farshbaf-Khalili A., Seyyedzavvar A., Fuladi N., Hosseini N., Talashi S. (2021). Short-term Outcomes of Launching Mother’s Milk Bank in Neonatal Intensive Care Unit: A Retrospective Study. Arch. Iran. Med..

[26] Torres-Munoz J., Jimenez-Fernandez C.A., Murillo-Alvarado J., Torres-Figueroa S., Castro J.P. (2021). Clinical Results of the Implementation of a Breast Milk Bank in Premature Infants (under 37 Weeks) at the Hospital Universitario del Valle 2018–2020. Nutrients.

[27] Taiwan Society of Neonatology. http://www.tsn-neonatology.com.

[28] Brown N.C., Doyle L.W., Bear M.J., Inder T.E. (2006). Alterations in neurobehavior at term reflect differing perinatal exposures in very preterm infants. Pediatrics.

[29] Lin Y.C., Chen Y.J., Huang C.C., Shieh C.C. (2020). Concentrated Preterm Formula as a Liquid Human Milk Fortifier at Initiation Stage in Extremely Low Birth Weight Preterm Infants: Short Term and 2-year Follow-up Outcomes. Nutrients.

[30] Calgary U.O. Research Bulk Calculator for Gestational Age Size at Birth. https://live-ucalgary.ucalgary.ca/resource/preterm-growth-chart/calculators.

[31] Jobe A.H., Bancalari E. (2001). Bronchopulmonary dysplasia. Am. J. Respir. Crit. Care Med..

[32] International Committee for the Classification of Retinopathy of Prematurity (2005). The International Classification of Retinopathy of Prematurity revisited. Arch. Ophthalmol..

[33] Neu J., Walker W.A. (2011). Necrotizing enterocolitis. N. Engl. J. Med..

[34] Kantorowska A., Wei J.C., Cohen R.S., Lawrence R.A., Gould J.B., Lee H.C. (2016). Impact of Donor Milk Availability on Breast Milk Use and Necrotizing Enterocolitis Rates. Pediatrics.

[35] Leaf A., Dorling J., Kempley S., McCormick K., Mannix P., Linsell L., Juszczak E., Brocklehurst P., Abnormal Doppler Enteral Prescription Trial Collaborative Group (2012). Early or delayed enteral feeding for preterm growth-restricted infants: A randomized trial. Pediatrics.

[36] Herrmann K., Carroll K. (2014). An exclusively human milk diet reduces necrotizing enterocolitis. Breastfeed. Med..

[37] Abiramalatha T., Thanigainathan S., Ninan B. (2019). Routine monitoring of gastric residual for prevention of necrotising enterocolitis in preterm infants. Cochrane Database Syst. Rev..

[38] Stey A., Barnert E.S., Tseng C.H., Keeler E., Needleman J., Leng M., Kelley-Quon L.I., Shew S.B. (2015). Outcomes and costs of surgical treatments of necrotizing enterocolitis. Pediatrics.

[39] Fengler J., Heckmann M., Lange A., Kramer A., Flessa S. (2020). Cost analysis showed that feeding preterm infants with donor human milk was significantly more expensive than mother’s milk or formula. Acta Paediatr..

[40] Ganapathy V., Hay J.W., Kim J.H. (2012). Costs of necrotizing enterocolitis and cost-effectiveness of exclusively human milk-based products in feeding extremely premature infants. Breastfeed. Med..

[41] Delfosse N.M., Ward L., Lagomarcino A.J., Auer C., Smith C., Meinzen-Derr J., Valentine C., Schibler K.R., Morrow A.L. (2013). Donor human milk largely replaces formula-feeding of preterm infants in two urban hospitals. J. Perinatol..

[42] Shenker N., Virtual Collaborative Network of Human Milk Banks and Associations (2020). Maintaining safety and service provision in human milk banking: A call to action in response to the COVID-19 pandemic. Lancet Child Adolesc. Health.

[43] Merjaneh N., Williams P., Inman S., Schumacher M., Ciurte A., Smotherman C., Alissa R., Hudak M. (2020). The impact on the exclusive breastfeeding rate at 6 months of life of introducing supplementary donor milk into the level 1 newborn nursery. J. Perinatol..

