# Supplementary material for: Growth of Very Preterm Infants in a Low-Resourced Rural Setting after Affiliation with a Human Milk Bank
Source: Children (Basel). 2022 Jan 5;9(1):80. doi: 10.3390/children9010080 (PMC8774553; doi:10.3390/children9010080)
Supplement: Supplementary file 1 [file children-09-00080-s001.zip › children-1514181-supplementary.pdf]

Table S1. Mini reviews: Summarized benefits of the implementation of human milk bank in neonatal intensive care units.

Authors	Years	Results
Arslanoglu, S., et al. [22]	2013	Exclusive breastfeeding rate at discharge was significantly higher in NICUs with a HMB.
Sparks, H., et al. [23]	2018	Calorie intake increased markedly during the first two weeks.
Alyahya, W., et al. [24]	2019	DHM helps preterm neonates in accordance with existing local guidance.<br>Using DHM as first milk feed did not affect subsequent MOM availability.
Hosseini, M., et al. [25]	2021	Improved the outcomes of premature infants (NEC, ROP, LOS).
Torres-Munoz, J., et al. [26]	2021	Improved the outcomes of premature infants (NEC, IVH, sepsis).

NICU: neonatal intensive care unit; HMB: human milk bank; DHM: donor human milk; MOM: mother's own milk; NEC: necrotizing enterocolitis; ROP: retinopathy of prematurity; LOS: late onset sepsis; IVH: intraventricular hemorrhage. [number]: reference number in the main text]

Table S2. Characteristics and novelties of Taiwan Southern Human Milk Bank.

**Location**	**Nation Cheng Kung University Hospital, Tainan, Taiwan**
Website 1	https://human-milk-bank.org/
Website 2	http://milkbank.hosp.ncku.edu.tw/
Facebook	https://www.facebook.com/giveumilk
Annual budget	USD$ 20,000
Funding source	National Health Service, Ministry of Health and Welfare, Taiwan
Annual processing volume of donor human milk	2 million – 3 million mL raw milks
Novelties	Case management for each donor; volunteers for long-term donation and cooperation; rejection of a single batch donation; door to door to serve donors with sterile bottle and to transfer collected raw human milks.
Participated donor per year	120–150
Qualified donors per year	100–120
Affiliated distribution sites	3 medical centers; 9 rural or local hospitals.
Service for personal recipients requiring pasteurized donor human milk	Yes; approved after the evaluation of physicians in the milk bank
Total served infants per year	600–900
Contact or accessing method	Email; Line®; QR-code with Google Sheet®; Facebook messenger®; No fax

Table S3. Dependence of body weight at term equivalent age on clinical variables (univariate analysis and multivariate analyses).

	**Univariate**			**Multivariate Model 1**			**Multivariate Model 2**			**Multivariate Model 3**		
	**B**	**95%CI (LB, UB)**	***p***	**B**	**95%CI (LB, UB)**	***p***	**B**	**95%CI (LB, UB)**	***p***	**B**	**95%CI (LB, UB)**	***p***
Gestational age	0.005	(-0.057, 0.067)	0.879	0.058	(0.005, 0.111)	**0.034**	0.056	(0.003, 0.108)	**0.037**	0.047	(-0.007, 0.101)	0.086
Z-score of birth weight	0.287	(0.135, 0.439)	**<0.001**	0.315	(0.167, 0.462)	**<0.001**	0.310	(0.165, 0.456)	**<0.001**	0.317	(0.171, -0.462)	**<0.001**
Sex	0.358	(0.102, 0.615)	**0.007**	0.408	(0.190, 0.627)	**<0.001**	0.389	(0.173, 0.605)	**0.001**	0.375	(0.158, 0.592)	**0.001**
Multi-gestational pregnancy	0.373	(0.008, 0.738)	**0.045**				0.271	(-0.027, 0.569)	0.074	0.252	(-0.048, 0.551)	0.098
PDA ligation	-0.411	(-0.838, 0.016)	0.059							-0.215	(-0.595, 0.165)	0.262
Epoch (ref. Epoch I)	0.355	(0.103, 0.607)	**0.006**	0.236	(0.014, 0.459)	**0.038**	0.224	(0.005, 0.443)	**0.045**	0.185	(-0.044, 0.414)	0.111

Linear regression was performed for each dependent variable and independent variable. Statistical significance was assumed for $p < 0.05$ (indicated in bold). PDA: patent ductus arteriosus; B: mean of coefficients; CI: confidence interval; LB: lower border; UB: upper border.

Table S4. Dependence of Z-score of body weight at term equivalent age on clinical variables (univariate analysis and multivariate analyses).

	**Univariate**			**Multivariate Model 1**			**Multivariate Model 2**			**Multivariate Model 3**		
	**B**	**95%CI (LB, UB)**	***p***	**B**	**95%CI (LB, UB)**	***p***	**B**	**95%CI (LB, UB)**	***p***	**B**	**95%CI (LB, UB)**	***P***
Gestational age	0.077	(-0.047, 0.202)	0.220	0.196	(0.095, 0.296)	**<0.001**	0.193	(0.094, 0.293)	**<0.001**	0.182	(0.078, 0.286)	**0.001**
Z-score of birth weight	0.710	(0.422, 0.999)	**<0.001**	0.800	(0.521, 1.080)	**<0.001**	0.804	(0.528, 1.080)	**<0.001**	0.809	(0.531, 1.086)	**<0.001**
Sex	0.192	(-0.355, 0.740)	0.486	0.356	(-0.061, 0.773)	0.093	0.325	(-0.089, 0.738)	0.122	0.306	(-0.112, 0.724)	0.149
Multi-gestational pregnancy	0.560	(-0.191, 1.311)	0.141				0.466	(-0.104, 1.036)	0.108	0.441	(-0.135, 1.017)	0.131
PDA ligation	-0.786	(-1.654, 0.083)	0.075							-0.277	(-1.006, 0.452)	0.450
Epoch (ref. Epoch I)	0.813	(0.310, 1.316)	**0.002**	0.536	(0.111, 0.961)	**0.014**	0.510	(0.089, 0.931)	**0.018**	0.461	(0.019, 0.903)	**0.041**

Linear regression was performed for each dependent variable and independent variable. Statistical significance was assumed for $p < 0.05$ (indicated in bold). PDA: patent ductus arteriosus; B: mean of coefficients; CI: confidence interval; LB: lower border; UB: upper border.

Table S5. Dependence of ΔZ-score of body weight at term equivalent age on clinical variables (multivariate analyses).

	**Multivariate Model 4**			**Multivariate Model 5**		
	**B**	**95%CI (LB, UB)**	***p***	**B**	**95%CI (LB, UB)**	***p***
Gestational age	0.210	(0.104, 0.315)	**<0.001**	0.177	(0.065, 0.288)	**0.002**
Z-score of birth weight	-0.096	(-0.382 0.189)	0.503	-0.128	(-0.408, 0.153)	0.367
Sex	0.356	(-0.071, 0.783)	0.100	0.386	(-0.042, 0.813)	0.076
Multi-gestational Pregnancy	0.461	(-0.119, 1.041)	0.117	0.455	(-0.123, 1.033)	0.121
Late onset sepsis	-0.127	(-0.774, 0.520)	0.695			
Bronchopulmonary dysplasia				-0.259	(-0.784, 0.265)	0.326
Epoch (ref. Epoch I)	0.405	(-0.086, 0.896)	0.104	0.415	(-0.027, 0.857)	0.065

Linear regression was performed for each dependent variable and independent variable. Statistical significance was assumed for $p < 0.05$ (indicated in bold). B: mean of coefficients; CI: confidence interval; LB: lower border; UB: upper border.
